# Iodine Nutrition and the Prevalence of Thyroid Disease after Salt Iodization: A Cross-Sectional Survey in Shanghai, a Coastal Area in China

**DOI:** 10.1371/journal.pone.0040718

**Published:** 2012-07-27

**Authors:** Shurong Zou, Fan Wu, Changyi Guo, Jun Song, Cuihua Huang, Zhenni Zhu, Huiting Yu, Yanfei Guo, Xi Lu, Ye Ruan

**Affiliations:** 1 Department of Nutrition Hygiene, Shanghai Municipal Center for Disease Control and Prevention, Shanghai, China; 2 Department of Health Statistics, Shanghai Municipal Center for Disease Control and Prevention, Shanghai, China; 3 Department of Environmental Health, Shanghai Municipal Center for Disease Control and Prevention, Shanghai, China; 4 Department of School Health, Shanghai Municipal Center for Disease Control and Prevention, Shanghai, China; 5 Department of Diabetes Mellitus Control and Prevention, Shanghai Municipal Center for Disease Control and Prevention, Shanghai, China; Cardiff University, United Kingdom

## Abstract

**Background:**

Both insufficient and excess iodine may produce thyroid disease. After salt iodization in China, the median urine iodine concentration (UIC) of children aged 8–10 years appeared adequate. However, it is unknown whether dietary changes due to rapid economic development in Shanghai have affected whole population iodine nutrition.

**Objective:**

To assess dietary iodine intake, UIC and the prevalence of thyroid disease in the general population of Shanghai.

**Design:**

A cross-sectional survey was conducted with general participants aged 5–69 years (n = 7,904) plus pregnant and lactating women (n = 380 each) selected by stratified multistage sampling. The iodine concentrations in their salt, drinking water and urine were measured. Daily iodine intake was estimated using the total diet study approach. Serum thyroid hormone concentrations and thyroid-related antibodies were measured and thyroid ultrasonography was performed.

**Results:**

The median iodine concentration in salt was 29.5 mg/kg, and 12.8 µg/L in drinking water. Iodized salt, used by 95.3% of participants, contributed 63.5% of total dietary iodine. Estimated daily iodine intake was 225.96 µg. The median UIC of general participants was 146.7 µg/L; UIC <100 µg/L (iodine insufficiency) was seen in 28.6%; UIC >300 µg/L (iodine excess) in 10.1%. Pregnant women had a median UIC of 135.9 µg/L, with UIC <150 µg/L in 55.4%. Thyroid nodules and subclinical hypothyroidism were found in 27.44% and 9.17%, respectively.

**Conclusions:**

According to published criteria, the current dietary iodine intake in Shanghai was generally sufficient and safe, but insufficient in pregnant women. Thyroid nodules and subclinical hypothyroidism were the commonest thyroid diseases identified.

## Introduction

Iodine is required for the synthesis of thyroid hormones; both insufficient and excess intake may lead to thyroid disease. Data from the World Health Organization (WHO), United Nations Children’s Fund (UNICEF), and International Council for the Control of Iodine Deficiency Disorders (ICCIDD) in 2007 estimated that about 30% (1900.9 million) of the world’s population has insufficient iodine intake, with the worst affected regions according to the WHO being South-East Asia and Europe [1]. Iodine deficiency disorders (IDD) were once common in China. Therefore, a universal salt iodization (USI) program has been carried out in China since 1995, with effective control of IDD. An assessment from the Chinese Ministry of Health in 2000 indicated that China had completely eliminated IDD.

Shanghai, the biggest coastal city in China, had been considered a non-IDD-endemic area. However, a survey in 1995 of urine iodine concentration (UIC) in children aged 8–10 years indicated their concentration of nutritional iodine was low, with the median UIC of children from urban and suburban areas being 72.27 µg/L and 57.23 µg/L, respectively [2]. Therefore, iodized salt has been supplied in Shanghai since 1996, and the median UIC of children aged 8–10 years in Shanghai has been adequate or sufficient from 1997 to 2005 [3]–[6].

Rapid economic development in Shanghai has now introduced remarkable variations in diet and eating habits that may have influenced iodine intake, but whether iodine nutrition has actually changed as a result is unknown. Furthermore, a survey on children aged 8–10 years only cannot represent the nutritional iodine status of the entire population because other vulnerable groups in the population may have a different nutritional iodine status [7].

Therefore, the aim of this survey was to assess dietary iodine intake, UIC and the prevalence of thyroid disease in the whole population of Shanghai 13 years after the introduction of salt iodization. The information obtained could provide the basis for further government policy-making.

## Subjects and Methods

### Subjects

A cross-sectional survey was conducted from September 2009 to December 2009. All residents who had been in Shanghai for more than 12 months were initially eligible for inclusion. Those with serious mental disorders or dementia, those who were deaf or bedridden, or those who had hepatitis (infectious period), active tuberculosis, AIDS or other infectious diseases were excluded.

A multi-stage stratified random sampling scheme was used to recruit participants from general population. Firstly, two streets/towns were randomly selected from each district in Shanghai. Secondly, two neighborhoods/villages were randomly selected from each selected street/town. Thirdly, a number of resident groups were chosen from each selected neighborhood/village to meet the sample size requirements. In the fourth stage, 13 children/adolescents for each age group (5–9, 10–14, 15–19 years) and 65 households for participants aged 20–69 years were randomly recruited from selected resident groups in each neighborhood/village; the KISH table was used to determine an adult participant aged 20–69 year from each of the 65 households.

Ten pregnant women and ten lactating women were randomly recruited from records registered from August 1 to August 31, 2009, in the above selected streets/towns from each district, respectively.

A multi-stage sampling method was also used based on above recruited unites for dietary iodine assessment. Firstly, ten districts were randomly selected from all districts. Secondly, one neighborhood/village was randomly selected from the above selected neighborhoods/villages in each of the ten districts. Finally, 30 households from the 65 selected households in the above selected neighborhood/village were randomly recruited for dietary assessment. The potential samples and response rates were listed in **Table 1** **.** The survey protocol was approved by the medical ethics committee of Shanghai municipal center for disease control and prevention. Written informed consent was obtained from all participants.

**Table 1:** A summary of the samples collected and investigations performed in the general population and the two subgroups.

Population	Sample	Number of potential samples	Number of responses/samples obtained (%)	Number of satisfactory samples/investigations (%)
	Table salt*	8664	7940/91.64	7617/87.92
	Drinking water*	8664	8106/93.56	8080/93.26
General participants	Questionnaire	7904	7410/93.75	7369/93.23
	Urine	7904	7113/89.99	6905/87.36
	Blood	5928	5228/88.19	5168/87.18
	Ultrasound	5928	5225/88.14	5167/87.16
	Dietary survey	300	279/93.00	279/93.00
Pregnant women	Urine	380	367/96.58	343/90.26
Lactating women	Urine	380	370/97.37	353/92.89

* If two participants were from the same household, only one set of table salt and drinking water samples was collected per household.

### Methods

A questionnaire was designed to obtain general personal information, which included sex, age, nationality, physical activity, personal or family history of thyroid disease (including time of diagnosis) and intake of iodine supplements. The questionnaire was administrated face-to-face by trained staffs in home. All questionnaires had been immediately checked for quality and completeness after the home interview. The total diet study approach was used to estimate the daily iodine intake for a “reference man”. The iodine concentrations in table salt, drinking water and urine were measured for all participants. Serum thyroid stimulating hormone (TSH), free triiodothyronine (FT3), free tetraiodothyronine (FT4), thyroglobulin antibody (TgAb), thyroid peroxidase antibody (TPOAb) and TSH receptor antibody (TRAb) were measured, and ultrasonography of the thyroid was performed for the general population aged over 15 years.

For each household, table salt samples of at least 100 g were collected, which were then sealed and stored at room temperature away from light until measurements could be made. The concentration of iodine was measured by the colorimetric titration method. The proportion of households using iodized salt was defined as the percentage of salt samples with an iodine concentration of at least 5 mg/kg. The proportion of households using adequately iodized salt was defined as the percentage of salt samples with an iodine concentration of 20–50 mg/kg (the current standard for salt iodization in China).

Each household also provided drinking water samples of at least 50 mL, which were then sealed and stored at room temperature away from light until measurements could be made using the spectrophotometric method.

The main processes of the total diet study approach were dietary survey, food clustering, sample collecting, food cooking and sample preparation. Food iodine concentration was measured using the inductively coupled plasma mass spectrometry method. To estimate the iodized salt contribution and iodine loss as a result of cooking, we prepared two sets of samples, one cooked with iodized salt, another cooked with noniodized salt.

All participants provided spot urine samples of at least 20 mL, which were sealed and stored at −20°C until measurements could be made using the colorimetric ceric ion-arsenious acid ash method, based on the Sandell–Kolthoff reaction.

Approximately 5 mL fasting venous blood was collected from the general population aged over 15 years. Serum was stored at −70°C after centrifugation until measurements could be made. TSH, FT3, FT4, TgAb and TPOAb were measured using the chemiluminescence immunoassay method, with electrochemical luminescence (Roche E601, Germany). TRAb was measured using radioimmunoassay.

Thyroid ultrasonography was performed by specially trained technicians using equipment with 7.5-MHz linear transducers.

### Diagnostic Criteria for Thyroid Diseases

The diagnostic criteria for thyroid diseases are listed in **Table 2** **** [**8**]–[**11**]. For the purposes of this study, patients were termed “euthyroid” if their thyroid gland was normal both by the blood assays and by ultrasound.

**Table 2:** Diagnostic criteria for the various thyroid diseases.

Thyroid Diseases	* Diagnostic Criteria
Nodule	
Single nodule	Normal thyroid volume with a single nodule >3 mm in diameter
Multiple nodules	Normal thyroid volume with >2 nodules >3 mm in diameter
Goiter	
Diffuse	Diffusely increased left and right lobes without nodules on ultrasound
Nodular	Asymmetrically increased left and right lobes, or no increased lobe size, on ultrasound. Irregular dark dense echoes and numerous nodules throughout the thyroid.
Hyperthyroidism	TSH <0.27 mIU/L, FT4>22 pmol/L orFT3>6.8 pmol/L
Subclinical hyperthyroidism	TSH <0.27 mIU/L, FT3 and FT4 within the normal range
Hypothyroidism	TSH >4.2 mIU/L, FT4<12 pmol/L
Subclinical hypothyroidism	TSH >4.2 mIU/L, FT4 within the normal range
Autoimmune thyroid disease	
Graves’ disease	Hyperthyroidism. Diffuse goiter on ultrasound. TPOAb >34 U/ml or TRAb ≥5 U/L
Chronic lymphocytic thyroiditis	
Hashimoto’s thyroiditis	Hypothyroidism. TPOAb >34 U/ml or TgAb >115 U/ml. Diffuse goiter on ultrasound without history of thyroid surgery or radioisotopic therapy
Atrophic thyroiditis	Hypothyroidism. TPOAb >34 U/ml or TgAb >115 U/ml. Thyroid atrophy on ultrasound without any history of thyroid surgery or radioisotopic therapy

* Reference ranges: FT3 3.1–6.8 pmol/L; FT4 12–22 pmol/L; TSH 0.27–4.2 mIU/L; TPOAb 0.0–34 U/ml; TgAb 0.0–115 U/ml; TRAb <5.00 U/L.

### Statistical Analysis

Statistical analysis was conducted by using SAS, version 9.1.3. Normally distributed data were expressed as the mean ± SD. Non-normally distributed data were expressed as the median, with the 25th and 75th percentiles. A comparison of proportion for different sex or age groups was performed using the Cochran–Mantel–Haenszel test. Comparison of UICs between euthyroid participants and those with thyroid disease was performed using the Wilcoxon test.

For undetected values, the processing was as follows: for values below the lower limit of detection (LOD), results were expressed as 1/2 lower LOD; for values above the upper LOD, results were expressed as 1/2 upper LOD [12], [13].

## Results

### Summary of Samples Obtained

The details of potential samples, the responses and samples obtained and the number of satisfactory samples for general population, pregnant women and lactating women are listed in **Table 1**.

Assessment of the representative features of current sample.

Myers’ index: that Myers’ index is greater than 60 in a sample means there is a serious age preference. However, Myer’s index was only 16.92 in the current whole sample (male: 17.04, female: 18.76). Therefore, our sample had no age preference (**Table 3**).

**Table 3:** Myers indexes of age heaping.

	Age terminal digit	5–59 yrs		15–69 yrs		*AX* + *BY*	Proportion (%)	|Proportion (%)−10%|
		Samplesize *A*	Weight*X*	Samplesize *B*	Weight*Y*			
Male +Female	0	620	1	508	9	5196	8.45	1.55
	1	549	2	464	8	4796	7.80	2.20
	2	569	3	481	7	5040	8.19	1.81
	3	579	4	515	6	5454	8.87	1.13
	4	543	5	462	5	5065	8.23	1.77
	5	750	6	625	4	6942	11.29	1.29
	6	709	7	640	3	6903	11.22	1.22
	7	671	8	589	2	6550	10.65	0.65
	8	790	9	597	1	7707	12.53	2.53
	9	785	10	602	0	7860	12.78	2.78
	Total					61513	100.00	16.92
Male	0	319	1	252	9	5192	8.44	1.56
	1	289	2	242	8	4810	7.82	2.18
	2	284	3	237	7	5074	8.25	1.75
	3	297	4	273	6	5406	8.79	1.21
	4	276	5	222	5	5025	8.17	1.83
	5	405	6	322	4	7000	11.38	1.38
	6	386	7	345	3	6883	11.19	1.19
	7	352	8	308	2	6546	10.65	0.65
	8	411	9	294	1	7707	12.53	2.53
	9	402	10	309	0	7850	12.77	2.77
	Total					61493	100.00	17.04
Female	0	301	1	256	9	2587	8.13	1.87
	1	260	2	222	8	2514	7.90	2.10
	2	285	3	244	7	2511	7.89	2.11
	3	282	4	242	6	2826	8.88	1.12
	4	267	5	240	5	2490	7.82	2.18
	5	345	6	303	4	3718	11.68	1.68
	6	323	7	295	3	3737	11.74	1.74
	7	319	8	281	2	3432	10.78	0.78
	8	379	9	303	1	3993	12.55	2.55
	9	383	10	293	0	4020	12.63	2.63
	Total					31828	100.00	18.76

Comparison of age distribution of our sample with whole population of Shanghai in the end of 2008: we compared our sample with the whole population of Shanghai in end of 2008 through chi-squared test. There were differences of age distribution when all sampled participants were compared to the whole population, but no differences when only those aged 20 yrs or over were compared (**Table 4** **,** **5**). This may be due to over sampling for those aged 5–19 yrs. We, therefore, used the age of the whole population in Shanghai to adjust estimated iodine status.

**Table 4:** Goodness of fit test of age distributions of current sample (aged 5–69 yrs) and population in Shanghai in 2008.

Age group (yrs)	Population in Shanghai (%) Pi			Sample (%) Si			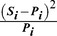		
	Total	Male	Female	Total	Male	Female	Total	Male	Female
5∼	2.94	2.96	2.92	13.14	13.80	12.42	35.39	39.70	30.91
10∼	3.40	3.39	3.40	12.46	12.46	12.45	24.14	24.27	24.09
15∼	5.51	5.47	5.56	11.20	11.49	10.88	5.88	6.63	5.09
20∼	8.49	8.51	8.46	4.33	4.21	4.46	2.04	2.17	1.89
25∼	9.48	9.56	9.39	4.59	4.79	4.37	2.52	2.38	2.68
30∼	7.38	7.45	7.32	5.20	4.97	5.44	0.64	0.83	0.48
35∼	7.31	7.30	7.32	4.60	4.63	4.57	1.00	0.98	1.03
40∼	7.76	7.83	7.69	6.23	6.63	5.80	0.30	0.18	0.46
45∼	11.29	11.40	11.17	8.64	8.76	8.52	0.62	0.61	0.63
50∼	14.06	14.06	14.06	10.60	10.26	10.96	0.85	1.03	0.68
55∼	10.63	10.36	10.91	8.12	7.97	8.27	0.59	0.55	0.64
60∼	7.23	7.24	7.22	6.61	6.15	7.09	0.05	0.16	0.00
65∼	4.53	4.48	4.58	4.30	3.89	4.74	0.01	0.08	0.01
Chi-square value							74.05	79.56	68.60

Degree of freedom: 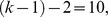 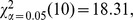 All 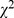 values were greater than cut-off points in the whole, female and male samples.

**Table 5:** Goodness of fit test of age distributions of current sample (aged 20–69 yrs) and population in Shanghai in 2008.

Age group (yrs)	Population in Shanghai (%) Pi			Sample (%) Si			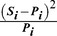		
	Total	Male	Female	Total	Male	Female	Total	Male	Female
20∼	9.63	9.65	9.61	6.85	6.76	6.94	0.83	0.87	0.74
25∼	10.75	10.84	10.66	7.26	7.69	6.81	1.11	0.92	1.39
30∼	8.38	8.44	8.30	8.22	7.98	8.47	0.00	0.03	0.00
35∼	8.29	8.28	8.30	7.28	7.44	7.11	0.11	0.09	0.17
40∼	8.80	8.88	8.72	9.85	10.65	9.04	0.11	0.35	0.01
45∼	12.80	12.93	12.68	13.68	14.07	13.27	0.06	0.10	0.03
50∼	15.95	15.95	15.96	16.77	16.48	17.07	0.05	0.02	0.08
55∼	12.06	11.75	12.38	12.84	12.8	12.88	0.04	0.09	0.02
60∼	8.20	8.21	8.19	10.46	9.89	11.04	0.60	0.34	0.99
65∼	5.14	5.08	5.19	6.81	6.25	7.38	0.54	0.27	0.92
Chi-square value							3.45	3.07	4.36

Degree of freedom: 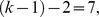 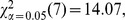 All 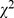 values were greater than cut-off points in the whole, female and male samples.

The gender ratio of the sample and whole population in Shanghai: The male to female ratio was 1.07 (male: 3803, female: 3566) in our sample, similar to the sex ratio in the corresponding age range of the whole population in Shanghai (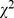 = 1.87,P = 0.1713).

### The Iodine Concentrations in Table Salt and Drinking Water

The median iodine concentration in table salt was 29.5 mg/kg (25th–75th percentiles: 26.2–32.7 mg/kg). The proportion of households using iodized salt was 95.3%, and the proportion of households using adequately iodized salt was 91.5%. The median iodine concentration in the drinking water was 12.8 µg/L (25th–75th percentiles: 7.4–14.2 µg/L).

Iodine supplementation was taken by 74 participants (0.92%), who each took 146.6±44.2 µg daily. The percentage of participants with a personal or family history of thyroid disease was 4.98% (402 cases).

### The Daily Iodine Intake for a Reference Man

A reference man was defined as an 18-year-old man with low physical activity. As shown in **Table 6** **,** iodine concentration in aquatic products, eggs and products, vegetables, meat and poultry was relatively high. The estimated daily salt intake for a reference man was 6.72 g, and the daily iodine intake from diet was 225.96 µg.

**Table 6:** Iodine concentration and the daily iodine intake for a reference man from 12 kinds of food.

Food sorting	Iodine concentration (mg/kg)	The daily iodine intake for a reference man (µg/d)
Cereals and products	0.005	2.66
Legumes and products	0.193	15.74
Tubers and products	0.109	2.08
Meat and poultry	0.213	22.93
Eggs and products	0.333	11.68
Aquatic products*	0.420	24.99
Milk and products	0.184	18.46
Vegetables	0.249	123.54
Fruits	0.000	0.00
Sugars	0.000	0.00
Beverages and water	0.004	3.61
Liquor	0.011	0.28
Total	–	225.96

* Aquatic products: fish, shellfish, molluscs, kelp and seaweed.

The daily iodine intake for a reference man when food was cooked with iodized salt was 225.96 µg, but 82.55 µg if food was cooked with noniodized salt. Based on the above data, the iodized salt contributed 63.5% of the total dietary iodine, whilst cooking caused the loss of 24.6% of the iodine from the iodized salt. Fish, shellfish and molluscs cooked with noniodized salt contributed 13.8% of the total dietary iodine, with 40.7% in kelp and seaweed. Fish, shellfish and molluscs cooked with iodized salt contributed 5.03% of the total dietary iodine, with 14.9% in kelp and seaweed.

### Urine Iodine Concentration

The median UIC of general population aged 5–69 years was 146.7 µg/L (25th–75th percentiles: 92.0–215.9 µg/L). The proportions with a UIC <100 µg/L (the cutoff for iodine insufficiency) and >300 µg/L (the cutoff for iodine excess) were 28.6% and 10.1%, respectively; the proportions with a UIC in the range 100–199 µg/L (adequate iodine nutrition) and in the range 200–299 µg/L (above requirements) were 41.6% and 19.7%, respectively.

The median UIC of the male general population was 150.0 µg/L (25th–75th percentiles: 95.4–221.6 µg/L) and of the female general population was 141.7 µg/L (25th–75th percentiles: 89.6–211.3 µg/L). The median UIC in the different age groups are listed in **Table 7**; all were in the range 100–199 µg/L (adequate iodine nutrition), but there was a decreasing trend with age.

**Table 7:** The urine iodine concentration (UIC) in the general population aged 5–69 years.

Age (years)	n	Median (25th,75th percentiles) (µg/L)
5∼	927	166.5 (103.2, 259.2)
10∼	867	175.5 (111.3, 250.7)
15∼	721	148.5 (98.2, 212.7)
20∼	576	146.6 (98.3, 207.8)
30∼	674	146.3 (88.7, 222.1)
40∼	1031	137.7 (90.6, 199.4)
50∼	1339	133.0 (84.1, 195.9)
60∼	770	129.1 (79.7, 200.8)
8∼10	700	167.8 (110.2, 260.4)

The median UIC of the pregnant women was 135.9 µg/L (25th–75th percentiles: 81.6–195.3 µg/L); 55.4% of them had a UIC of <150 µg/L (the cutoff for iodine insufficiency). The median UIC of the lactating women was 131.1 µg/L (25th–75th percentiles: 76.4–191.0 µg/L); 36.8% of them had a UIC of <100 µg/L (the cutoff for iodine insufficiency).

### The Prevalence of Thyroid Disease

Abnormal serum TSH, FT3 and FT4 concentrations were found in 10.95%, 1.2% and 4.06% of the general population aged over 15 years. The percentage of female participants with an abnormal TSH concentration was higher than that of the male participants (13.32% vs. 8.64%, *P*<0.001), while the opposite was true for FT4 concentration (3.41% vs. 4.7%, *P* = 0.018). No significant difference was found between the sexes in the percentage of participants with an abnormal FT3 concentration. The percentage of participants aged over 15–39 years with an abnormal FT4 concentration was higher than that for participants aged 40–69 years. There were no significant differences across the age groups in the percentages of participants with abnormal TSH and FT3 concentrations.

Positive TPOAb, TgAb and TRAb concentrations were found in 8.46%, 10.29% and 0.93% of participants aged over 15 years. Women were more likely to be positive for TPOAb and TgAb than men (TPOAb 11.12% vs. 5.85%; TgAb 15.12% vs. 5.58%; *P*<0.001). No significant difference was found between the sexes in the percentage with a positive TRAb level (1.07% vs. 0.78%, *P* = 0.2822). There were no significant differences across the age groups in the percentages with positive TPOAb, TgAb and TRAb concentrations.

The prevalence of diffuse goiter, nodular goiter, single nodule and multiple nodules were 0.72%, 1.38%, 15.56% and 11.88%, respectively. The prevalence of goiters (diffuse and nodular) and thyroid nodules (single and multiple) were 2.1% and 27.44%, respectively, as shown in **Table 8**. Women had a higher prevalence of thyroid nodules than men (33.32% vs. 21.62%; *P*<0.001); no significant difference was found in the prevalence of goiters between men and women. The prevalence of thyroid nodules and goiters in participants aged 40–69 years was higher than in those aged 15–39 years (thyroid nodule 33.57% vs. 18.34%; goiter 2.92% vs. 1.01%; *P*<0.001).

**Table 8:** The prevalence of various thyroid diseases in the general population aged 15–69 years.

	Gender		Age (years)		
Thyroid diseases	Male (%)	Female (%)	15–39 (%)	40–69 (%)	Total (%)
Goiter	1.94	2.24	1.01	2.92^1^	2.10
Thyroid nodules	21.62	33.32^2^	18.34	33.57^1^	27.44
Hyperthyroidism	0.50	0.94	0.71	0.72	0.72
Subclinical hyperthyroidism	0.27	0.26	0.05	0.41^1^	0.27
Hypothyroidism	0.44	0.98^2^	0.46	0.85	0.71
Subclinical hypothyroidism	7.3	11.03^2^	8.44	9.66	9.17

1 *P*<0.05 compared with participants aged 40–69 years (Cochran–Mantel–Haenszel test).

2 *P<*0.05 compared with women (Cochran–Mantel–Haenszel test).

The prevalence of hyperthyroidism, subclinical hyperthyroidism, hypothyroidism and subclinical hypothyroidism were 0.72%, 0.27%, 0.71% and 9.17%, respectively (**Table 8**). Women had a higher prevalence of hypothyroidism and subclinical hypothyroidism than men (hypothyroidism 0.98% vs. 0.44%, *P* = 0.0166; goiter 11.03% vs. 7.30%, *P*<0.001); no significant differences were found between the sexes in the prevalence of hyperthyroidism and subclinical hyperthyroidism. The prevalence of subclinical hyperthyroidism in participants aged 40–69 years was however higher than those aged 15–39 (0.41% vs. 0.05%, *P* = 0.0164), but there were no significant differences across the age groups in the prevalence of hyperthyroidism, hypothyroidism or subclinical hypothyroidism.

The prevalence of Graves’ disease and chronic lymphocytic thyroiditis were 0.21% and 0.31%, respectively.

The comparison of UICs between euthyroid participants and those with thyroid disease are listed in **Table 9**. Participants with multiple nodules had lower UICs than euthyroid participants (129.28 µg/L vs. 143.28 µg/L, *P*<0.001); those with hyperthyroidism had higher UICs than euthyroid participants (195.42 µg/L vs. 143.28 µg/L, *P* = 0.001). No significant differences in UICs were found between euthyroid participants and those with a single nodule, goiter, subclinical hyperthyroidism, hypothyroidism or subclinical hypothyroidism.

**Table 9:** Comparison of urine iodine concentrations (UICs) between euthyroid participants and those with thyroid disease.

Group		n	Median (25th,75th percentiles) (µg/L)
Thyroid nodules	Single	775	139.46 (81.49, 204.80)
	Multiple	610	129.28 (76.82, 190.11)^1^
Goiter	Diffuse	36	136.23 (82.28, 191.13)
	Nodule	70	131.06 (87.41, 215.15)
Hyperthyroidism*		22	195.42 (154.02, 280.10)^1^
Subclinical hyperthyroidism*		14	138.94 (87.64, 201.58)
Hypothyroidism		36	128.82 (81.90, 179.02)
Subclinical hypothyroidism		467	144.46 (92.40, 216.3)
Euthyroid participants#		2567	143.28 (91.75, 210.2)

* As patients with hyperthyroidism or subclinical hyperthyroidism were not allowed to take iodine or foods with high iodine content by recommendation of their doctors, participants with a known history of hyperthyroidism or subclinical hyperthyroidism are excluded from this table.

# A euthyroid participant was defined as one whose thyroid gland was normal both by the blood assays and by ultrasound.

1 *P*<0.05 compared with the normal population, (Wilcoxon test).

## Discussion

Our results estimated that the daily iodine intake of a reference man was 225.96 µg, using the total diet study approach. The recommended dietary allowance (RDA) of iodine for an adult man is 150 µg/d, according to the WHO/UNICEF/ICCIDD [14]; the upper daily tolerable intake level of iodine is 1000 µg/d according to the Chinese Nutrition Society [15]; the tolerable daily intake (TDI) of iodine is 600 µg/d according to the WHO [16]. Based on these criteria, the current dietary iodine intake in Shanghai is sufficient and safe.

In this survey, the iodized salt contributed 63.5% of the total dietary iodine, while the aquatic products, which are considered rich in iodine, contributed 5.03% of the total dietary iodine, with 14.9% in laver and kelp, which was markedly lower than the contribution of iodized salt. As indicated above, salt was the main source of iodine intake; the consumption of table salt and the iodine concentration within it are the main factors that influence iodine intake. Our results indicated a median table salt concentration of 29.5 mg/kg, which was within the range of China’s current iodized salt standard.

The UIC, when carried out with appropriate technology and sampling, is currently the most practical biochemical maker for iodine nutrition. Our results indicated the median UIC of general population aged 5–69 years was 146.7 µg/L, and 135.9 µg/L and 131.1 µg/L in pregnant and lactating women, respectively. Based on current diets, the proportion of households using iodized salt and adequately iodized salt was >90%. Based on the median UICs, according to the criteria from WHO/UNICEF/ICCIDD 2007 for assessing iodine nutrition [17], iodine nutrition was adequate in the general population and in lactating women, but insufficient in pregnant women. The iodine requirement during pregnancy is increased because of fetal demand. Pregnant women are particularly vulnerable to iodine deficiency because of the harmful effect of deficiency for normal growth and neurodevelopment of the fetus. Pregnant women have also been found to be deficient in iodine in several previous studies [18]–[20]. Therefore, more attention must be given to iodine intake during pregnancy.

The association between the iodine intake of a population and the occurrence of thyroid disease in that population is U-shaped. There is a relatively narrow range for optimal intake; disease is more likely to develop in the populations with intakes above and below this range. Our survey found the prevalence of thyroid nodules and subclinical hypothyroidism in Shanghai were 27.44% and 9.17%, respectively, these being the two commonest types of thyroid disease, based on currently sufficient iodine intake and adequate iodine nutrition.

Thyroid nodules are common in clinical practice; increasing age, female sex, iodine deficiency, and a history of head and neck radiation increase the risk of thyroid nodules [21]–[23]. In clinical practice a variety of thyroid diseases exist in nodular form; the use of high-resolution ultrasound (HRUS) increases detection of nodules. In addition to the method of screening, their prevalence depends largely on the population being evaluated: in non-goiter-epidemic areas, the prevalence of thyroid nodules was 2–6% with palpation [24]–[29], 19–34.7% with ultrasound [26], [30], [31] and 8.2–65% from autopsy data [32]–[34]. Our results using ultrasound found that the prevalence of thyroid nodules was 27.44%; higher in women than men, and that prevalence increased with age. The median UICs of participants with a single nodule and multiple nodules were 139.46 µg/L and 129.28 µg/L respectively, which were adequate. However, compared with euthyroid participants, those with multiple nodules had a lower UIC, and there was a trend toward decreased concentrations found in participants with a single nodule, which suggests the UIC of the population may be one factor that influences the occurrence of thyroid nodules.

The reported prevalence of subclinical hypothyroidism differs from 1% to 20% [35]. Diagnostic criteria (the reference range for serum TSH and FT4), race, age, sex, and iodine nutrition are the main factors that influence the prevalence of subclinical hypothyroidism. Studies from Spain, Italy, Japan and North America have reported the prevalence of subclinical hypothyroidism as 3.2–13% [36]–[38], based on serum TSH >4.0 mU/L as the diagnostic criteria. In our survey, the prevalence was 9.17%, being higher in women. No difference in UIC was found between euthyroid participants and those with subclinical hypothyroidism.

The sample size in our survey was designed on the basis of the UIC of the population, so there may be a bias in the prevalence of thyroid disease because of insufficient sample size. Therefore, our discussion does not focus on those thyroid diseases with a prevalence below 8%.

Since the introduction of iodized salt, we have been monitoring the nutritional iodine status based on the UIC and the prevalence of goiter in children aged 8–10 years, which should not be used to represent the nutritional iodine status of the entire population. Therefore, our survey provides baseline data for further studies that consider dietary iodine intake, the UIC in the general population and certain special populations, and the prevalence of thyroid disease.

In conclusion, the current dietary iodine intake in Shanghai is sufficient and safe. Iodine nutrition was generally adequate, but insufficient in pregnant women. Thyroid nodules and subclinical hypothyroidism were the two commonest types of thyroid disease.
